# Persistent Organic Pollutants: Environmental Stain Fading Fast

**DOI:** 10.1289/ehp.115-a20

**Published:** 2007-01

**Authors:** Tim Lougheed

Shocked that chemical offshoots of his company’s products were winding up in polar bears and seals, the CEO of 3M put an end to the existing version of Scotchgard® in 2000. Business observers were struck by how quickly this multinational corporation removed a flagship commodity worth hundreds of millions of dollars from the market. Now some scientific observers are becoming impressed by how quickly the degradation products that prompted this move appear to be disappearing from the environment.

University of Toronto chemist Scott Mabury has been among those identifying a decline in the presence of agents such as perfluorooctane sulfonate (PFOS), one of a class of manufactured chemicals that feature chains of carbon atoms bonded to fluorine atoms. In an *Environmental Science & Technology* paper published online 30 November 2006, Mabury and colleagues report a significant drop in PFOS levels in Arctic ringed seals over the last two years to about a third of what they were in 2000, along with ongoing declines in levels of related fluorine-based compounds.

Mabury notes that change has followed hard on the heels of 3M’s withdrawal of its old version of Scotchgard, which consisted of precursors to PFOS and other perfluorinated carboxylic acids, part of a class of perfluorinated chemicals (PFCs). PFCs have come under fire since their ubiquity and persistence were confirmed in the late 1990s, although their toxicological potential as carcinogens and hormone disruptors is still being investigated. PFOS and its cousin perfluorooctanoic acid (PFOA) have been found in blood samples from humans living all over the world and animals in Arctic, temperate, and tropical regions.

For several decades, PFCs had found widespread use in applications such as nonstick coatings for metal and paper surfaces, as well as stain and water repellants on textiles and carpeting. After its accidental discovery in the early 1950s, Scotchgard became one of the best known and most popular of these products, appealing to consumers who wanted to keep wine stains off their upholstery and an even more substantial industrial client base with highly technical goals, such as optimizing the performance of microchips.

John Giesy, now the holder of a Canada Research Chair in Environmental Toxicology at the University of Saskatchewan and the researcher who first reported PFCs in the environment, was with Michigan State University in 1997 when 3M asked him to offer opinions on the state of the knowledge of compounds produced by the company. This led to joint research projects to answer specific questions such as whether PFOS, the major degradation product of many of the company’s chemistries, was present in the general environment.

Working together with 3M, Giesy and colleague Kurunthachalam Kannan had soon analyzed several thousand samples from remote locations and confirmed the presence of PFOS in the environment. The first mass spectrometry systems capable of such analysis on fluorine—incorporating innovations such as electrospray and triple quadrupole design—were just becoming available, and 3M helped Giesy take advantage of these developments by providing methods and access to instruments.

Giesy vividly recalls the climax of these efforts, when he was invited to bring his findings directly to 3M officials, who subsequently reported the information to the EPA and announced they would voluntarily cease essentially all production of PFOS-containing products. “They were very courageous, they made the right decisions,” he says, noting that because he had been able to investigate the toxicity of other PFCs, he was able to help the company swiftly reformulate Scotchgard in a way that drastically minimized the release of PFCs.

Such efforts are shaping regulatory regimes internationally. At the start of 2006, following an expert advisory panel report citing PFOA as a likely carcinogen, the EPA began inviting fluoropolymer and telomer manufacturers to join the agency’s new stewardship program on this and related chemicals. The participants—eight major firms including 3M/Dyneon, Ciba Specialty Chemicals, and E.I. duPont de Nemours & Company—formally committed to reducing PFOA from emissions and product content by 95% by 2010, with the ultimate goal of eliminating PFOA from emissions and products by 2015.

Meanwhile, Canada, which in 2004 placed a two-year ban on four fluorinated polymers containing telomer alcohols, recently proposed extending this measure to a permanent ban on the manufacturing, sale, and importation of these chemicals. Nevertheless, this proposal does not cover consumer products containing these chemicals, which may be imported from other markets.

As for PFOS, the Stockholm Convention on Persistent Organic Pollutants proposed a global ban on this and closely related substances in June 2005. Sweden and the United Kingdom had earlier taken national bans on PFOS to the European Commission, urging a European Union–wide ban. That drive resulted in a proposed directive to restrict the use of PFOS in carpets, textiles, clothing, and other items, which is expected to go into effect in the next couple of years.

To Mabury, these efforts can only benefit from the relatively rapid results being seen in affected environments, even as he continues to explore the processes behind those results. “This first data set suggests a very rapid response in a remote environment near the top of the food chain, from an industrial change happening just a few years before,” he says. “I view that as a fabulously good news story.”

## Figures and Tables

**Figure:**
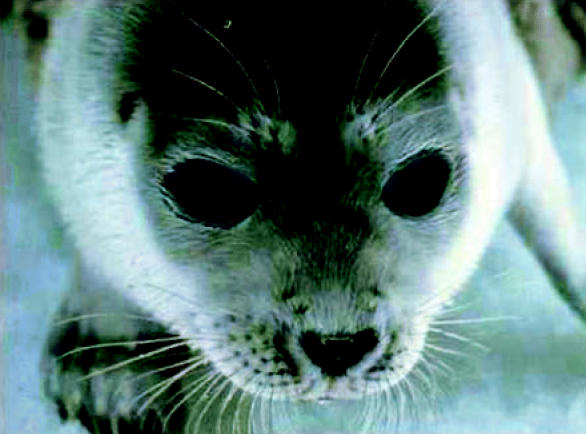
Creating a bright spot. The reformulation of Scotchgard has led to rapidly declining levels of PFCs in the Arctic environment and in the bodies of animals including the ringed seal.

